# Allergy prevention—a new public health perspective

**DOI:** 10.1093/eurpub/ckaf258

**Published:** 2025-12-31

**Authors:** Tari Haahtela, Pekka Puska

**Affiliations:** Department of Clinical Allergology, University of Helsinki, Helsinki, Finland; Director General Emeritus, Finnish Institute for Health and Welfare (THL), Helsinki, Finland

Allergy is a rapidly growing public health problem leading to suffering and societal costs, especially in the Western countries but also globally [1]. In connection with the health collaboration between North Karelia, Finland, and Russian Karelia attention was drawn also to the big differences in allergy between these two areas with contrasting environments and lifestyles. Finns had a strikingly higher prevalence of allergy and asthma than the population on the Russian side [2]. There was also a remarkable cohort effect. Among adults born in the 1940s there was no difference in the objectively measured sensitization against pollen, but among those born in 1980s the difference was manifold.

The reasons for the development were not genetic, air pollution or common environmental chemicals but evidently contrasting exposure to biodiverse environment—also a surrogate marker of the lifestyle—which associated with skin microbiome and immune regulation [3]. This observation was the basis for the biodiversity hypothesis of allergic disease and the subsequent studies.

Subsequent research supporting this hypothesis has, indeed, pointed out the role of nature connection—i.e. connection with microbes of the soil, natural waters and air. Studies have shown how green environment around homes are inversely associated with allergic symptoms and how children grown in the countryside with cattle and pets have less allergy [4].

The microbiome of the urban citizens has significantly impoverished compared to our ancestors [5]. The environmental exposures throughout life drives the adaptive epigenetic machinery supervising the protein synthesis. In addition to the microbes also environmental biogenic chemicals play a role.

This research has called for a new approach to prevent and manage allergy and asthma: turning avoidance into tolerance and to emphasize allergy health instead of disease. Results of this work in Finland have been most encouraging [6]. With greatly reduced disease burden major saving in direct and indirect healthcare costs have been observed.

This emerging information is being applied in many European countries. In 2019, the European Patient Association (EFA) started the Awareness Campaign for allergy, asthma, and COPD, and in 2022, EU included the Finnish Allergy Programme to the Best Practice Portal. In many countries, e.g. France, Germany, Lithuania, Netherlands, Norway, Poland, Portugal, Sweden, UK actions have been taken at the population level to improve allergy prevention, treatment, and self-management. In Portugal, a media campaign has commenced to advice people with allergy about changes in their life.

The action under slogan “Nature step” has been implemented in Finland in one city of Lahti and in a local health service area. Next year, the campaign is implemented nationally. The indicators for the follow-up are asthma, diabetes, obesity and depression.

We have long recognized various risk factors for chronic noncommunicable diseases. Interventions to control these factors have been successful. However, there is another side of the coin, namely protective factors [4]. It is apparent that our health is a result of balance of risk and protective factors. Cultural evolution, built on biological evolution, has provided safety, innovations, scientific breakthroughs, better healthcare and prolonged life expectancy. Urbanization is aimed for better life, but increasingly disconnecting humankind from the wider nature, his evolutionary roots. Human body is an ecosystem dependent on the wider ecosystems.

It is possible that allergic symptoms—immediate worsening by pollen, animal dander, food—are signaling much larger but slowly emerging public health problems characterized by microbial dysbiosis, immune imbalance, and low-grade inflammation. They may also be relevant for various noncommunicable diseases like diabetes.

From the public health perspective, benefits of improving nature connection in prevention and management of allergic disorders and noncommunicable diseases in general seems to be plausible. The hard evidence is still scanty as controlled interventions are few, but with new results and experiences there are promises not only for public health but also for broader benefits.

We are not going back to nature, but natural elements can be taken back to urban environment and to everyday life. Profound changes are needed in lifestyle and behavior, societal structures and green economy. The good news is that such actions for public health contribute to the urgent need to mitigate global warming and stop nature loss. Nature positivity and nature-based solutions are required to support health, environment and economy in the urban world. In 2019, the European Commission advanced Nature-based solutions as central to biodiversity, climate neutrality and resilience.

Conflict of interest: None declared.
